# Utility of Common Marmoset (*Callithrix jacchus*) Embryonic Stem Cells in Liver Disease Modeling, Tissue Engineering and Drug Metabolism

**DOI:** 10.3390/genes11070729

**Published:** 2020-06-30

**Authors:** Rajagopal N. Aravalli, Clifford J. Steer

**Affiliations:** 1 Department of Electrical and Computer Engineering, College of Science and Engineering, University of Minnesota, 200 Union Street S.E., Minneapolis, MN 55455, USA; 2 Departments of Medicine and Genetics, Cell Biology and Development, University of Minnesota, 420 Delaware Street S.E, Minneapolis, MN 55455, USA; steer001@umn.edu

**Keywords:** liver, marmoset, *Callithrix jacchus*, embryonic stem cell, transplantation, hepatocyte, liver disease, regeneration, drug metabolism, cell therapy, cytochrome P450, bioengineering

## Abstract

The incidence of liver disease is increasing significantly worldwide and, as a result, there is a pressing need to develop new technologies and applications for end-stage liver diseases. For many of them, orthotopic liver transplantation is the only viable therapeutic option. Stem cells that are capable of differentiating into all liver cell types and could closely mimic human liver disease are extremely valuable for disease modeling, tissue regeneration and repair, and for drug metabolism studies to develop novel therapeutic treatments. Despite the extensive research efforts, positive results from rodent models have not translated meaningfully into realistic preclinical models and therapies. The common marmoset *Callithrix jacchus* has emerged as a viable non-human primate model to study various human diseases because of its distinct features and close physiologic, genetic and metabolic similarities to humans. *C*. *jacchus* embryonic stem cells (cjESC) and recently generated cjESC-derived hepatocyte-like cells (cjESC-HLCs) could fill the gaps in disease modeling, liver regeneration and metabolic studies. They are extremely useful for cell therapy to regenerate and repair damaged liver tissues in vivo as they could efficiently engraft into the liver parenchyma. For in vitro studies, they would be advantageous for drug design and metabolism in developing novel drugs and cell-based therapies. Specifically, they express both phase I and II metabolic enzymes that share similar substrate specificities, inhibition and induction characteristics, and drug metabolism as their human counterparts. In addition, cjESCs and cjESC-HLCs are advantageous for investigations on emerging research areas, including blastocyst complementation to generate entire livers, and bioengineering of discarded livers to regenerate whole livers for transplantation.

## 1. Introduction

Global mortality rates due to liver disease are approximately two million, annually. This includes one million deaths caused by complications due to cirrhosis and the remaining owing to viral hepatitis and liver cancer [1]. The liver plays a major role in many key functions, including glucose metabolism, bile production, drug metabolism, glycogen and vitamin storage, and the production of coagulation factors. It is also the site where many inherited and acquired genetic disorders occur as well as for the treatment of certain inborn errors of metabolism that do not directly cause injury to the liver [2]. For some, the only viable therapeutic option for acute and chronic liver failure is orthotopic liver transplantation (OLT). Even though liver transplantation is the second most common transplantation procedure worldwide, the availability of suitable livers for transplantation is low and not in keeping with the demand [1]. To overcome this problem, cell transplantation with primary human hepatocytes (PHH) is being used to bridge the gap to OLT in patients with acute and chronic liver disease. Hepatocytes comprise about 80% of the total liver cell mass and perform key functions, such as metabolic homeostasis, bile synthesis, detoxification and storage of vitamins. They also carry out critical functions by metabolizing carbohydrates, fats, proteins and drugs. Various enzymes produced in hepatocytes regulate excess glucose and synthesize it if needed, oxidize triglycerides to produce energy, convert excess carbohydrates and proteins into fatty acids and triglyceride, synthesize lipoproteins, phospholipids, non-essential amino acids, cholesterol, urea and glycogen, and produce most plasma proteins such as albumin [3,4,5,6,7]. Pluripotent stem cells that are able to differentiate into all liver cell types and could efficiently engraft into the liver parenchyma are also ideal candidates for cell transplantation.

Different types of liver tissues, such as whole or split livers from organ donors, and discarded liver sections during therapeutic interventions, are the sources of PHHs. The quality and functionality of these hepatocytes is dependent on the characteristics of the donor livers, including gender, age, health or pathological status, and previous drug treatments. Unfortunately, the limited availability of suitable donor liver tissue to isolate hepatocytes, poor quality of hepatocytes isolated from discarded cadaveric livers, inability of adult hepatocytes to survive beyond a few passages in vitro, and difficulties to fully replicate their enzymatic functions in experimental conditions have been major challenges for their use in therapeutic applications [8]. As a result, there is a scarcity in obtaining sufficient numbers of high-quality PHHs for populating the diseased liver to restore its functions. This has led to the identification of alternative sources of hepatocytes with greater reliability, proliferation, and differentiation into multiple liver cell types, such as pluripotent stem cells, from species that closely recapitulate human liver functions. To this end, stem cell-derived hepatocyte-like cells (HLC) not only provide a continuous source of cells for transplantation, but also are potentially useful for disease modeling, and investigations into drug function and metabolism.

Over the years, efforts have been made to isolate, generate and culture cell lines from livers with the purpose of using them for various clinical applications, biochemical and pharmacological studies, and for cell transplantation. While a few cell lines were isolated from carcinogen-treated or choline-deficient rodents as well as from liver tumors, others were obtained by immortalizing primary hepatocytes. Bipotential liver stem cell lines that could differentiate into two principal liver cell types (hepatocytes and cholangiocytes) were isolated from mice treated with retrorsine and subsequent partial hepatectomy [9], and from albumin-urokinase plasminogen activator/severe combined immunodeficiency disease transgenic mice [10]. Successful establishment of bipotential progenitor cell lines by overexpressing the simian virus 40 (SV40)-encoded large T antigen (TAg) in fetal epithelial liver cells from cynomolgus monkeys was also reported [11]. However, rodents often present difficulties in accurately presenting the disease under study as well as predicting the response to treatment in humans [12,13,14]. These differences are manifested particularly in immune functions, epigenetic regulation and disease pathogenesis, including that of the liver [15,16,17,18,19]. Therefore, non-human primates (NHP) that provide a more conducive environment for human hepatocyte engraftment and stem cell proliferation and differentiation are preferable models to study cell-based therapeutics. In addition, primary NHP hepatocytes and stem cells from NHPs are appropriate model systems to investigate human liver disorders.

## 2. The Common Marmoset Is an Ideal NHP Model for Research on Liver Diseases

**Figure 1:**
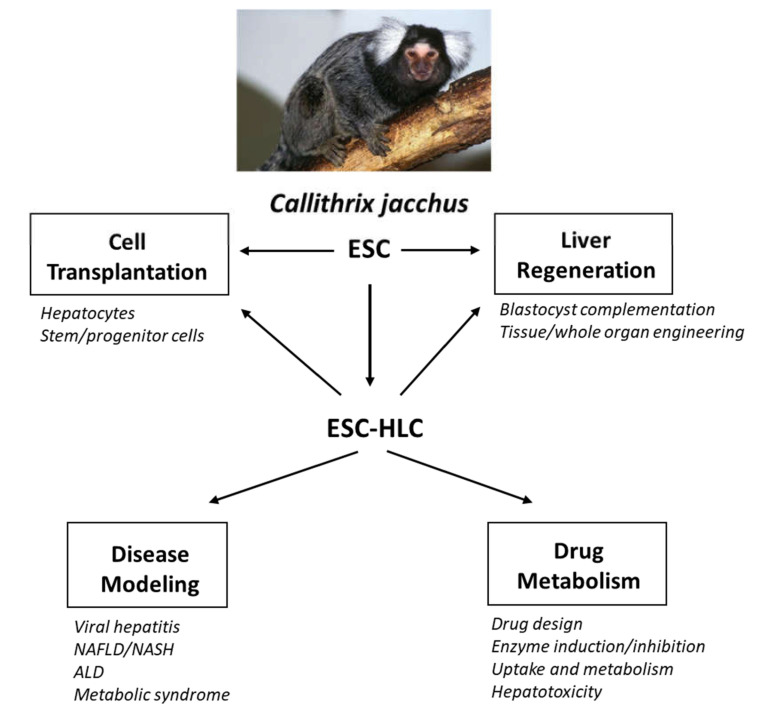
Potential uses for *C. jacchus* ESC and ESC-HLC in liver research.

The common marmoset (*Callithrix jacchus*) is a “New World Monkey” native to Atlantic coastal forests of northeastern Brazil [20,21]. It has become a viable NHP model for many human diseases, including those of the liver [12,21,22,23,24,25,26,27,28], because of its unique characteristics. Various studies have also shown that *C. jacchus* closely mimics human diseases and physiological conditions, such as neurodegenerative disorders, reproductive biology, spinal cord injury, stroke, infectious disease, behavioral research, drug development and safety assessment [21,22,26,29]. Adult marmosets have an average height of 20–30 cm, weight of 350–400 grams and a shorter life span (10 to 15 years). Small body size, shorter gestation period (~144 days), ease of handling, established animal husbandry techniques, and lower maintenance costs than other NHPs, such as rhesus macaque and cynomolgus monkeys (two commonly used “Old World Monkeys”), make them suitable for biomedical research [21,24,27,28,30,31]. Since they reach sexual maturity by 18 months of age and frequently give birth to twins or triplets, rapid expansion of existing marmoset colonies can be achieved. Marmosets have proven to be much closer to humans for pharmacokinetic and toxicological screening than rodents [32,33], and their cells effectively cross-react with human cytokines and hormones [21,27]. Moreover, they are not known to carry any endogenous viruses that are harmful to humans [21], and manifest fewer zoonotic diseases than Old World monkeys [22]. The relative liver mass of marmosets is similar to that of humans, making it an ideal animal model to study common liver diseases, such as non-alcoholic fatty liver disease (NAFLD) [31] and hepatitis C virus (HCV) infection [34]. In addition, marmosets are appropriate models for drug metabolism and toxicological studies because of their expression of key metabolic enzymes, such as the cytochrome P450 superfamily, which is similar to that of humans [23,24] (Figure 1).

## 3. Marmoset Embryonic Stem Cells

Embryonic stem cells (ESC) are pluripotent stem cells that are capable of differentiating into all three germ layers. They possess enormous potential to self-renew indefinitely and develop into all types of cells and tissues in the body. These characteristics make ESCs ideal for studies on disease modeling, tissue engineering, organ regeneration, production of transgenic animals, and drug development. Since the isolation and establishment of mouse cultures in 1981 [35,36], ESCs have been isolated from many mammalian species and were successfully differentiated in vitro into various therapeutically relevant cell types [37]. The first set of eight common marmoset embryo–derived pluripotent stem cell lines were isolated in 1996 [38]. Subsequently, other research groups also established *C. jacchus* ESC (cjESC) cell lines [39,40,41,42]. Studies have shown that they can be propagated in vitro both on feeder layers and in feeder-independent culture conditions [43,44], and that they can be genetically modified using CRISPR/Cas9 gene editing and the PiggyBac transposase system [45,46]. Moreover, they can be converted from the primed to a naive-like state using transgenes to increase their pluripotency in vitro [47]. cjESCs were recently differentiated into highly functional hepatocyte-like cells (cjESC-HLCs) [48], which would be valuable for in vitro studies on infectious diseases, regenerative medicine and drug metabolism. While it has been shown that iPSCs can allograft into the putamen of cynomolgus monkeys without immunosuppression [49], cjESC-derived cells were only tested using immunosuppressive agents such as tacrolimus [50]. However, it was reported that marmoset ESCs do enable allograft or autograft transplantations in the absence of immunosuppressive agents, presumably in other marmosets, and thus may facilitate a more precise assessment of the safety and efficacy of stem cell transplantation [51]. In summary, cjESCs provide important research tools for basic and applied research that could not be carried out with human ESCs (hESC) due to ethical and moral considerations.

## 4. *Callithrix jacchus* Models of Human Liver Disease

The common marmoset is an appropriate NHP model for studying various liver diseases because of its close proximity to humans in physiology, genetics, and immunology. Many studies have shown that it is susceptible to human viruses and could recapitulate human disease conditions. In light of these findings, both *C. jacchus* and cjESCs have become indispensable for preclinical research to evaluate safety and effectiveness of drug candidates, and for studies on infectious diseases and regenerative medicine. In addition, the use of cjESCs could overcome certain limitations of human iPSCs for disease modeling, including incomplete reprogramming of mature cells, high cell-to-cell and line-to-line variability, confounding effects of cell culture, lack of standardization of methods to confirm pluripotency and differences from adult cell physiology [52,53,54].

### 4.1. Viral Hepatitis

In humans, viral hepatitis is caused by infection with one of five hepatotropic viruses, which include hepatitis A virus (HAV), hepatitis B virus (HBV), HCV, hepatitis D virus (HDV) and hepatitis E virus (HEV) [55]. An ideal animal model for any hepatitis virus infection should mimic all the relevant clinical features as observed in humans. Preferably, it should be susceptible to all viral genotypes with resulting persistent viremia [19]. However, progress on developing a reliable animal model has been hampered by the extremely narrow host range of these viruses, which typically do not infect rodent hepatocytes. Therefore, transgenic mouse models that express the whole genome or individual genes of HBV or HCV, and humanized mice that express various human factors such as CD81, OCLN, CLDN1 and SR-BI and infected with hepatotropic viruses have been developed to study viral hepatitis [56,57,58,59]. As the mouse immune system tolerates transgenetically expressed viral proteins, infection develops in the absence of both liver inflammation and fibrosis [60]. Even though rodent models of hepatitis virus have provided enormous amounts of data on these viruses, they have severe limitations since they exhibit immunodeficiencies.

The cloning of HCV genome from a chimpanzee that was infected with non-A, non-B hepatitis [61] has shed light on NHPs as useful animal models for viral hepatitis, which eventually resulted in the development of vaccines against HAV and HBV infections. In fact, chimpanzees are most suitable hosts for hepatitis viruses A–D studies [62,63,64]. But their use in experimental research is severely restricted as they are endangered species, controversial with animal rights advocates, and due to the high costs involved [63]. During the past three decades, different strains of HAV have been adapted in primates and primate cells in vitro, including *C. jacchus* [65,66]. Marmosets were suitable to model HAV infection because human HAV could infect them as shown in an earlier study. Using a mixture of in vitro transcribed cDNA clone of HAV strain HM-175 and full-length genomic RNA transcript, HAV was injected directly into the marmoset livers [67]. Animals that received this mixture developed acute hepatitis and the elevation of liver enzymes isocitrate dehydrogenase (IDH), alanine aminotransferase (ALT) and γ-glutamyl transpeptidase (GGT), which correlated with the appearance of antibodies to the HAV capsid proteins. The HAV was then isolated from the marmoset and was shown to originate from the cDNA clone that was injected into these animals. Liver biopsies further demonstrated that the cDNA encoded a virulent HAV and the histopathological changes were similar to those of wild-type HAV [67]. Marmosets with HAV infection showed an increase in inducible nitric oxide synthase expression, which correlated with an increase in the numbers of splenic CD2^+^ T-lymphocytes, and necrotic inflammatory lesions in the liver [68]. In other studies, *C. jacchus* infected intravenously with two different strains of HAV developed antibodies against HAV and expressed hepatitis A antigen in the liver [69]. In subsequent studies, marmosets were shown to develop acute hepatitis when infected with a Brazilian strain HAF-203 of HAV [70,71]. Collectively, these experiments also underscored the susceptibility of *C. jacchus* to human HAV. In addition to hepatitis viruses, marmosets are vulnerable to infection with other human viruses [72], which make them ideal animal models to study human infectious diseases. The reason for this high susceptibility of marmosets to infection with human viruses is unknown but limited diversity of their MHC Class I and II loci was thought to play a role [73,74].

In 1995, the GB virus-B (GBV-B) was discovered in tamarins that were injected with the serum of a human hepatitis patient [75]. It was subsequently shown that the virus could also infect marmosets [76]. GBV-B is a flavivirus closely related to HCV, and because of its structural, functional and genomic similarities with HCV, it can be used as a surrogate for HCV; and a *C. jacchus* model of GBV-B was subsequently developed [77,78]. In common marmosets, GBV-B induces T-cell and innate immune responses similar to that of HCV infection in humans [79,80]. The major difference is, in humans, HCV frequently establishes chronic infections whereas in marmosets, GBV-B causes only acute infections [76]. However, when two HCV/GBV-B chimeric viruses containing HCV structural genes coding for either the whole core and envelope proteins (CE1E2p7) or full envelope proteins (E1E2p7) were inoculated into the livers of marmosets, they developed hepatitis [81]. Pathological examination of liver tissues revealed lymphocyte infiltration, severe ground glass degeneration, cholestasis, eosinophilic cells, fibrous expansion, hepatic edema, cell disarray, and ultrastructural changes including abnormal mitochondrial, lipid droplets and increased numbers of lysosomes. A modest antibody response to HCV core and E2 proteins was also detected [81]. Marmosets infected with another HCV/GB-V chimeric virus expressing nonstructural proteins NS2 to NS4A of HSC also exhibited viremia with characteristics typical of viral hepatitis [82]. These findings indicated that common marmosets infected with chimeric viruses are valuable tools in the development of vaccines and antiviral drugs against HCV infection.

Many experimental systems currently exist for studying HCV in vitro using human hepatoma and HCC cell lines; and human fetal liver cells, which support HCV replication have been developed (reviewed in [83]). However, to study HCV infection with PHHs is a challenge because cultured adult human hepatocytes display only a low level of HCV infection when compared to these cell lines. Disease modeling with patient-derived clinical HCV isolates is also a major problem. To overcome these bottlenecks, successful attempts have been made to model HCV infection in HLCs isolated from human-induced pluripotent stem cells (iPSCs) [84,85] and human ESCs [85]. Since *C. jacchus* is a good surrogate for HCV, the common marmoset could be an appropriate animal model to investigate hepatitis viruses in in vitro studies; and recently generated cjESC-HLCs [48] could be very valuable tools for in vitro experiments.

### 4.2. Hepatic Fibrosis

Hepatic fibrosis (HF) is characterized by the decomposition of excess extracellular matrix (ECM), complex cellular interactions between activated HSCs that produce ECM and other hepatic as well as infiltrating cells. Further, it is an imbalance between ECM production and degradation, which leads to the development of a progressive fibrosis [86,87,88]. In humans, HF is caused by various drug, metabolic, inflammatory, toxin, congenital, parasitic and vascular stimuli [86]. At the molecular level, it is marked by aberrant activity of transforming growth factor-β1 and its downstream mediators [87]. In animal models, HF is normally induced with compounds such as carbon tetrachloride, diethylnitrosamine or thioacetamide (TAA), which are metabolized mainly by the cytochrome P450 (CYP) enzyme CYP2E1 in hepatocytes causing centrilobular liver damage [88].

To date, no animal model was able to recapitulate all the hepatic and extra-hepatic features of HF. In a recent study, HF was induced in the *C. jacchus* by injecting TAA 2 to 3 times a week subcutaneously for up to 9 weeks [89]. The animals that were administered with TAA showed rapid development of periportal fibrosis to a morphological and pathophysiological status resembling human HF [89]. Furthermore, serum and histological examination of liver biopsies taken between 3 and 9 week intervals showed the development of progressive HF with the expression of various known markers of HF, such as blood type IV collage 7S, bilirubin and bile acids [89]. More recently, another group also developed a marmoset model of HF using repeated injections of TAA with similar results [90]. This latter animal model reproduced the pathology of human liver cirrhosis including portal hypertension. Following the generation of HF, human iPSC-derived HLCs were transplanted into the fibrotic livers via the portal vein to evaluate them as a cell therapy option. These HLCs engrafted into the liver parenchyma efficiently and ameliorated the fibrosis, suggesting that they can be used to bridge the gap to OLT [90]. ESC-derived HLCs are probably better candidates than iPSC-HLCs because of increased safety in patients, reduced maturation both in vitro and in vivo to a distorted phenotype, and improved variability in the phenotype. As discussed above, primary marmoset hepatocytes and cjESC-HLCs are potentially ideal candidates for such studies.

### 4.3. Non-Alcoholic Fatty Liver Disease

Non-alcoholic fatty liver disease (NAFLD) is a major healthcare problem worldwide due to the prevalence of obesity, hyperlipidemia, metabolic syndrome and type 2 diabetes. NAFLD results from excessive fat accumulation in the liver in the absence of significant alcohol intake. It could progress into non-alcoholic steatohepatitis (NASH), a more aggressive form that affects 20–30% of NAFLD patients [91,92,93]. NASH is prevalent in patients with obesity, diabetes and metabolic syndrome. It characterized by tissue damage, chronic liver inflammation and fibrosis. Liver fibrosis often leads to cirrhosis and hepatocellular carcinoma (HCC), the most common form of liver cancer, causing significant morbidity and mortality. A number of rodent models have been generated to study NAFLD by feeding them with a large variety of modified diets [91]. However, they do not mimic all the salient features of the human disease. While steatosis is a common feature of these animal models and NASH does occur in them, progression to HF is not common.

In contrast, marmosets can develop hepatic steatosis and recapitulate the NAFLD as seen in humans [31]. Those housed at the New England Primate Research Center were recently found to have profound hepatic enlargement and histologic lesions that are indicative of NAFLD [31]. The captive environment in which there is greater availability of food and lower physical activity is very similar to modern human environments. In total, 33 of 183 marmosets housed in this breeding colony displayed hepatomegaly and 31 of them tested positive for Oil Red O staining, evidence of obesity and insulin resistance [31]. Sixteen marmosets also showed ballooning and hepatocellular regeneration indicative of NASH, increased Ki-67 immunopositive cellular proliferation, marked elevation of serum triglycerides, GGT, hepatic leakage of aspartate transaminase (AST) and ALT, and had high serum levels of iron. In addition, the common marmoset exhibited increased hepatocellular lipid accumulation and inflammation [31]. The natural onset of this syndrome in captive animals demonstrated that marmosets could function as NHP models to study the full spectrum of NAFLD. Increased adiposity that occurs through similar underlying physiological processes [94] as well as hepatic siderosis that arises as a consequence of iron overload were also observed in callitrichids [95].

Recently, a human in vitro perfusion model of NAFLD was described that utilized PHHs in a 3D platform [96]. In this experimental model, cells were grown in a medium that was high in glucose, insulin and fat equivalent to 600 μmol/L free fatty acids (FFA). PHHs were grown for up to 14 days and the fat content of hepatocytes was measured by a combination of Oil Red O staining and various biochemical assays. PHHs grown in this diet accumulated three times more fat than cells grown in regular medium, and expression of adipokines in them increased significantly, but that of CYP3A4 and CYP2C9 decreased [96]. Moreover, the exposure to pioglitazone and metformin reduced triglyceride accumulation in fat loaded cells, which suggested that this in vitro model is suitable for drug screening. Since *C. jacchus* hepatocytes and cjESC-HLCs can be propagated in high culture volumes to obtain large cell numbers, they could be adapted in such a 3D/spheroid platform to recapitulate the clinical features of human NAFLD.

### 4.4. Metabolic Syndrome

Metabolic syndrome (MetS) is associated with abdominal obesity, inflammation, insulin resistance/type 2 diabetes, dyslipidemia [97]; and the entire spectrum of NAFLD is also regarded as the hepatic manifestation of MetS. Hepatokines, proteins predominantly produced and secreted by the liver, play critical roles in MetS and they influence the development of hepatic steatosis [98]. Fetuin-A, fetuin-B, retinol-binding protein 4, fibroblast growth factor 21, selenoprotein P, sex-hormone-binding globulin, chemotaxin 2, and angiopoietin-related protein 6 are the key hepatokines that are linked to the induction of metabolic dysfunction and they constitute an association between hepatic steatosis and insulin resistance that could eventually lead to NAFLD [98,99]. Several *C. jacchus* models have been described for both spontaneous and experimentally induced obesity with hyperglycemia and hypertriglyceridemia. These marmoset models were generated with diverse agents, such as human adenovirus-36 (Ad-36) [100], GBV-B [101], adult high calorie diet [102], and exposure to glucocorticoids [103].

Captive populations of *C. jacchus* that were maintained in basic forms of caging and husbandry were found to become spontaneously obese with high triglyceride and low-density lipoprotein content, in addition to altered glucose metabolism [104]. The study suggested that marmosets are good candidates to develop accurate NHP models of obesity by exposing them to high calorie density diets. Subsequently, a marmoset model of obesity was developed by feeding the animals with high-fat or glucose-enriched diets for up to 52 weeks [102], which elevated their glycosylated hemoglobin levels in the blood as early as 16 weeks and persisted for up to 52 weeks. Animals in the glucose-enriched diet group also had an increase in their fat mass but the animals from another group that were fed with a high-fat diet had only a transient increase in fat mass that soon returned to basal levels [102]. This finding suggested that glucose-enriched diets would more rapidly induce obesity in the common marmoset than those of high fat. In the Ad-36-induced model of MetS, marmosets were infected via intranasal inoculation of with 5 × 10^5^ PFU of Ad-36. They gained substantial weight in the 28-week period of the study, and blood samples revealed Ad-36 antibodies in the liver, and elevated serum levels of cholesterol and triglycerides [100]. In another investigation, marmosets that were infected intravenously with GBV-B, had a significant increase in their insulin, glucagon and glucagon-like peptide 1 in the plasma, became hypertriglyceridemic and had up to a 10-fold increase in adipocytokines [101]. In the liver, cytoplasmic changes associated with steatosis were observed at 168 days post-infection in all infected animals ranging from mild to severe steatosis. Most infected animals showed acute hypoglycemia and lipid accumulation in hepatocytes indicating hepatic steatosis, and hepatomegaly [101]. In the experimental model of glucocorticoid-induced MetS, pregnant marmosets were given the synthetic glucocorticoid dexamethasone orally for one week during the early or late gestation [103]. The expression of 11β-hydroxysteroid dehydrogenase type 1 (11β-HSD1) in different body tissues of the offspring was examined at 4 and 24 months of age because increased adipose or hepatic 11β-HSD1 was previously implicated in the pathogenesis of obesity and the metabolic syndrome [105]. mRNA expression of 11β-HSD1 in the offspring of marmosets fed with dexamethasone during late gestation was significantly elevated, including in the liver, and this increase occurred well before the animals developed obesity or displayed clinical features of MetS [103]. In addition, mothers of these offspring showed enhanced hepatic activity of PEPCK1, the rate-limiting enzyme of gluconeogenesis, suggestive of increased hepatic glucose output [103]. However, these marmoset models may not be useful because of the rapid evolution rate of protein-coding sequences for insulin and insulin-like growth factor-1 in the New World Monkeys, which can cause reduced affinity to anti-insulin antibodies [106].

## 5. Potential Uses for Marmoset ESCs in Liver Regeneration and Tissue Engineering

The liver is the only organ in the human body that is capable of renewing itself to its entirety. It has been shown that, even after 70% removal of the natural tissue via partial hepatectomy (PH), this remarkable regenerative capacity is achieved due to the proliferation of hepatic cells (hepatocytes, cholangiocytes, macrophages, epithelial cells and hepatic stellate cells) and, under special circumstances, stem/progenitor cells and bone marrow cells that repopulate the liver [107,108]. After PH, hepatocytes are mobilized to divide rapidly to restore the liver size, and when the original size of the organ is reached, they stop dividing so that the regenerating liver is not overgrown [107]. In early experiments with rats, it was shown that one third of hepatocytes that remained in the liver after the PH could restore the original liver mass in 7–10 days in a process that required fewer than two rounds of replication [107,109]. Thus, the organ size, which constitutes approximately 5% of the total body weight [110], is restored in a tightly controlled manner. This process of tissue restoration also replaces damaged cells to preserve the architecture and function of the organ [111], where stem/progenitor cells are critical.

To date, a number of different types of hepatic stem/progenitor cells have been isolated from healthy and diethoxycarbonyl-1,4-dihydrocollidine-treated rodents or from animals that were fed with choline-deficient diets and treated with the agent 2-acetamidofluorene [112,113]. Human pluripotent hepatic progenitor cells (hepatic stem cells and hepatoblasts) (HPCs) are present in stable numbers in healthy individuals throughout the life [114,115], and were also found in the diseased livers of patients with severe hepatocellular necrosis, chronic viral hepatitis and chronic alcoholic liver disease (ALD) [116]. In addition, stem cells from extrahepatic sources, such as the bone marrow, could also contribute to the formation hepatic cells in the liver [117]. Together with ESCs and other pluripotent stem cells, including iPSCs, these stem/progenitor cells are capable of differentiating into most liver cell types and could restore liver functions. Moreover, stem cell-derived HLCs are extremely valuable not only for in vitro studies to assess hepatocyte-specific functions but also to repopulate the diseased livers.

### 5.1. Cell Transplantation

For many end-stage liver diseases, cell transplantation is preferred as a ‘bridge’ to OLT. It is a viable treatment option particularly for metabolic diseases, whereby therapeutic genes and missing gene products can be transferred into cultured hepatocytes from allogeneic donors prior to ex vivo gene therapy. The implanted cells could then correct the inherited and acquired metabolic disorders. Cell transplantation can be repeated in sequential transplantations without immunosuppression and can be performed in more than one recipient from a single donor [118].

#### 5.1.1. Hepatocyte transplantation

Starting with the first human transplantation in cirrhotic patients [119], hepatocyte transplantation has been in use to correct various abnormalities, such as the inherited disorders Crigler-Najjar syndrome type 1 (CN1) [120], and glycogen storage disease type 1a [121]. The aim of hepatocyte transplantation is to restore liver functions in patients using a minimally invasive procedure with limited cell numbers and to prolong the need to replace the entire organ. Considering the minimum liver mass needed for a patient’s survival is about 30%, ~ 7.5 × 10^9^ cells are required for these transplantations [122], and it has been a challenge to obtain sufficient numbers of high-quality PHHs for this work. To overcome this roadblock, hepatocytes from animal sources such as the pig, human immortalized cells, and human tumor cell lines, have been proposed as alternative sources of PHHs. Other notable obstacles for hepatocyte transplantation include limited numbers of liver tissue that are available as cell source, lack of clinical grade reagents, hypothermic storage of cells without losing viability, procedures to enhance engraftment in vivo, proliferation of donor cells in vitro, tracking or monitoring cells after transplantation, and the optimal immunosuppression protocols for transplant recipients [123]. Another concern is transplanted hepatocytes are generally not observed after 6–9 months and it is not clear if this is due to rejection, apoptosis, or other causes [118]. While the preclinical studies on hepatocyte transplantation were successful in animals, their translation into the clinic has been disappointing [8,124]. Hepatocyte transplantation requires a large number of cells, transplanted hepatocytes have lower engraftment and poor survival rates [120], and about 70% of them are trapped in the hepatic sinusoids due to their larger size (20–40 µm) causing portal hypertension [125].

#### 5.1.2. Stem Cell Transplantation

To overcome obstacles associated with hepatocyte transplantation, stem/progenitor cells that are small in size and could differentiate into almost all hepatic cells in vivo are the most suitable cell type. Endothelial progenitor cell transplantation was used to treat liver fibrosis in rat livers where they were anti-fibrotic and stimulated liver regeneration [126]. Autologous mesenchymal stem cells tested in randomized trials in cirrhotic patients with and without HCV infection were, however, unsuccessful [127,128]. cjESCs are an attractive alternative to human ESCs for therapeutic studies on liver cirrhosis, acute liver failure, and metabolic liver diseases because they could provide a limitless supply of differentiated hepatic cells for transplantation in animals, and could differentiate into all hepatic cell types in vivo.

To achieve success with stem cell transplantation, the implanted stem cells should engraft efficiently into the liver parenchyma and differentiate into hepatic cells. ESCs and iPSCs are ideal cell types for this purpose as they not only have the differentiation potential to convert into all cell types but could also be expanded in cell culture. While ESCs are available from GMP compatible sources [129], there are still manufacturing obstacles and questions on the completeness of functionality of iPSCs [8,130], such as cell engraftment and differentiation in vivo, and risk of tumorigenesis. Moreover, the scale-up of iPSCs and in vitro differentiation has proven to be a difficult challenge, resulting in the production of small percentages of cells that are needed for transplantation. 

### 5.2. Whole Liver Tissue Engineering

In recent years whole-organ bioengineering using discarded livers has been proposed as an alternative approach to generate fully functional livers for OLT [131,132]. This approach utilizes the decellularization of livers and recellularization with freshly isolated functional hepatic cells. Decellularization can be achieved by a variety of chemical, mechanical and physical techniques that preserves the original 3D architecture and microvascular network of the liver, including ECM, while removing the entire the cellular content [133,134]. The decellularized liver scaffolds are then repopulated with hepatic cells by perfusion seeding [135]. In a previous experiment, rat livers were decellularized using sodium dodecyl sulfate and recellularized by four perfusions each with 5 × 10^6^ primary rat hepatocytes through the portal vein [133]. The injected hepatocytes engrafted at the efficiency of 95.6% and the recellularized liver tested positive for the expression of albumin, urea, various CYP enzymes and uridine diphosphate glucuronosyltransferases 1 (UGT1) [133]. Subsequent experiments with pig [136,137], sheep [138], and human liver tissues [139,140] using primary hepatocytes [139], immortalized hepatocytes [136,137], mesenchymal stem cells [141], human cell lines [140], and liver progenitor cells [138] for recellularization have produced promising results. However, only a few have reported the transplantation of a recellularized liver into an experimental animal [133,137,140]. Surprisingly, these studies were not yet performed with NHPs. *C. jacchus* could potentially be an ideal source for organ transplantation in humans with development of a bioengineered liver. Moreover, cjESCs and cjESC-HLCs can be used to repopulate the decellularized livers. Since marmoset livers are small for human transplantations, decellularized porcine livers repopulated with marmoset hepatic cells are suitable for use in humans. However, prior to their use in human applications, these cells should be extensively evaluated for possible immunological barriers that could cause rejection of xenotransplants, potential for xenogeneic infections, and the likelihood of long-lasting marmoset–human cell chimerism.

### 5.3. Blastocyst Complementation

In recent years, one novel approach of organ generation that has received much attention is ‘blastocyst complementation’ [142,143,144]. This method has immense potential as it allows the generation of cells and entire organs of one animal species (donor) into another (recipient species). In this method, for example, human–animal chimeric organs can be generated by knocking out the key gene(s) required for liver development in the blastocyst of the recipient animal via gene editing. It is then injected with donor human pluripotent stem cells, such as embryonic stem (ES) cells or induced pluripotent stem cells (iPSCs) that express those critical genes. In such manner, an entire human liver can be generated in a pig for human transplantation. This approach requires few pluripotent stem cells to create an entire organ. Most importantly, all of the inductive cues needed to generate the appropriate organ are present within the developing blastocyst and fetal environment so that potentially any organ can be produced in animals. Blastocyst complementation not only allows the creation of an entire liver, albeit chimeric, but also to obtain hepatocytes that are entirely human in origin to carry out in vitro studies on drug metabolism, and for hepatocyte transplantation. Using this methodology, the entire pancreas of a rat was grown in a mouse [145], kidney in rodents [146], and pancreas in pigs [147]. More recently, pancreas, kidney and the liver were produced in pigs using this approach [148].

Earlier studies have shown that during development of a mouse embryo, the liver first appears as an outgrowth bud of proliferating endodermal cells in the ventral foregut on day 8 of gestation [149]. Beyond the induction stage, various transcription factors needed for endoderm patterning and organ development are expressed in the embryo. Among these, the hematopoietically expressed homeobox gene *HHEX* plays a pivotal role in liver development [150], where it functions both as a transcriptional repressor and activator [151,152]. During early embryogenesis, *HHEX* mRNA is detectable at embryonic age (E) 8.5 in the developing embryo, and in the gut endoderm that gives rise to the liver [150,153]. Analysis of *HHEX*-null embryos demonstrated that the initial formation of the liver bud does not require functional protein but it is essential for development beyond E9.5, suggesting that *HHEX* is required to promote growth and differentiation of the hepatoblast stage [150,153,154]. In a subsequent study, a chimeric mouse was created by injecting *HHEX*^−/−^ ES cells into *HHEX*^+/+^/GFP^+^ blastocysts [154]. This study also demonstrated that *HHEX*^−/−^ cells were selectively excluded from the developing liver, and that the older highly chimeric embryos were devoid of *HHEX*^−/−^ cells in the liver, providing an explanation for the embryonic lethality in mice caused by disruption of the *HHEX* gene [150,154,155,156]. By injecting *HHEX*^+/+^ human stem-like cells into *HHEX*^−/−^ mouse blastocysts, we could expect that a chimeric liver can be produced that contains only hepatic cells of human origin using this approach [148]. The ability to use blastocyst complementation of cells from one species to restore the development of organs in another suggests the feasibility of growing marmoset organs in other animals or vice versa within a short time period.

## 6. Drug Metabolism Studies with Marmoset ESC-Derived HLCs

Before releasing a new drug into the market, it has to be tested adequately in appropriate vitro cell culture systems and in animal models for its safety and efficacy. Liver is the site where more than 90% of all the drugs are metabolized. Therefore, liver-based testing platforms will allow for the accurate prediction of pharmacokinetic and toxicological properties of drugs during the early stages of their development, and in clinical trials. As the primary site of drug metabolism, the liver functions to detoxify and facilitate the excretion of xenobiotics by enzymatically converting lipid-soluble compounds into more water-soluble compounds [157].

In general, drug metabolism and biotransformation of xenobiotics is achieved through phase I reactions, phase II reactions, or both. Phase I reactions are carried out by the CYP enzymes, whereas the majority of phase II enzymes are transferases [158,159]. Most of these enzymes are produced primarily in the liver by hepatocytes, and even in cirrhotic livers of patients with HCV infection and ALD [160]. PHHs are the gold standard for many of the studies on potential drug uptake and metabolism, mechanisms of hepatotoxicity of drugs, inhibition and induction of drug-metabolizing enzymes, and interaction of these enzymes with xenobiotics. However, use of PHHs in these studies has several limitations. They include maintenance of hepatocyte phenotype, rapid loss of many liver-specific functions, redistribution of canalicular membrane proteins, loss of cell polarity and architecture including that of bile canaliculi. The deterioration of cell viability within several days under conventional culture conditions precludes the use of this system for long-term studies and measurement of drug excretion [161]. To overcome these problems, various in vitro human liver models were developed, such as supersomes, microsomes, cytosol, S9 fraction, hepatic progenitor/stem cell lines, ESCs, transgenic cell lines, primary hepatocytes from rodents, 3D and sandwich-culture systems, human hepatoma cell lines, liver slices, and perfused liver [161,162,163,164,165,166,167]. Among these, PHHs and microsomes from liver tissues are widely used in studies on drug metabolism and inhibition. Microsomes are fractionated microscopic particles isolated from liver homogenates that are rich in RNA and contain CYP enzymes, esterases, amidases, flavin-containing monooxygenases, and epoxide hydrolases [168]. For in vivo drug metabolism studies, a number of animal models have been used, including ‘humanized’ mice that were either genetically modified to express human CYP, arylamine N-acetyltransferase, and uridine 5’-diphospho-glucuronosyl- transferase enzymes, or were transplanted with PHHs [169,170].

### 6.1. Phase I Enzymes

#### Cytochrome P450 Enzymes

CYPs are a super family of heme-containing enzymes that function mainly in the liver but are also present in other organs. They function as monooxygenases and carry out phase I metabolism of drugs, chemicals and other xenobiotics. In the human liver there are at least 57 distinct CYP enzymes [171]. Of these, isoenzymes from the families CYP1, CYP2 and CYP3 are involved in the hepatic metabolism of approximately 75% of therapeutic drugs [172,173]. Detailed knowledge on the expression and phase I metabolism of CYP enzymes in disease states would be useful in the development of rational drug therapies. Many studies have shown the effects of liver disease on CYP enzyme expression, and also the involvement of CYPs in the pathogenesis of disease [174,175]. For instance, the expression of CYP1A2, CYP2E1 and CYP3A was found to be decreased in cirrhotic and HCC patients, together with an alteration in the clearance of drugs metabolized by CYP3A4 [176,177]. Similarly, the expression of various CYP enzymes was altered in patients with NAFLD and ALD [178,179].

To date, 36 *C. jacchus* CYP isoenzymes (cjCYP) have been identified and 24 of them, belonging to 1A, 2A, 2B, 2C, 2D, 2E, 3A, 4A, and 4F subfamilies, share a high degree of homology in the cDNA (>89%) and amino acid sequences (>85%) with corresponding human P450s [180]. Among these, CYP1A2, 2A6, 2B6, four 2C subfamily members (2C8, 2C18, 2C19 and 2C58), 2D6, 2D8, 2E1, 2J2, 3A4, 3A5, 4A11, 4F2, 4F12 and 7B1 are expressed in the marmoset liver [181,182,183,184,185,186,187,188,189,190,191]. Interestingly, *CYP1D1*, encoded by a pseudogene in human liver is also pseudogenized in *C. jacchus* due to an incomplete open reading frame [180]. Induction studies have shown similar patterns between the common marmoset and human CYP orthologs. For instance, the expression of *C. jacchus* CYP1A2 is greatly enhanced in the liver by treatment with 3-methylcholanthrene [181] and 2,3,7,8-tetrachlorodibenzo-*p*-dioxin [192]. In in vitro induction assays, using either the hepatocytes or liver microsomes from *C. jacchus*, found CYP1A1 and 1A2 to be induced strongly by treatment with β-naphthoflavone and omeprazole, [193]; 1A6 with phenobarbital and rifampicin [182]; 2B6 with phenobarbital [183]; 2E1 with isoniazid [186]; and 3A with phenobarbital [182].

Various studies have also demonstrated that the metabolism and substrate specificities of *C. jacchus* CYPs are analogous to those of human CYPs. For example, an antidepressant and smoking cessation drug bupropion and an anti-arrhythmic drug propafenone are hydrolyzed by the CYP2D6 enzyme in hepatic microsomes from the *C. jacchus* at levels similar to those of the human microsomes [194,195,196]. Similarly, both human and *C. jacchus* CYP enzymes metabolized the carcinogen aflatoxin B_1_ (AFB), a risk factor in the development of HCC, at comparable levels [197].

cjESC-HLCs were recently shown to express three CYP enzymes (CYP1A2, CYP2E1 and CYP3A4) at levels comparable to those of their human counterparts [48]. Among these, CYP1A2, which accounts for about 13% of all CYP450 enzymes in the liver, is a major enzyme that metabolizes both endogenous compounds such as melatonin, estradiol, bilirubin and arachidonic acid, as well as several clinical drugs, including analgesics and antipyretics [198]. CYP3A4 is involved in the metabolic oxidation of more than 50% of all drugs including acetaminophen [199]. CYP2E1 metabolizes low molecular weight solvents such as alcohol, toxic chemicals like chloroform and carbon tetrachloride, and environmental contaminants such as benzene and acrylamide [200,201]. This broad substrate specificity represents the basis for many clinically relevant ‘drug–drug’ interactions and *C. jacchus* is a key model for studying these metabolic interactions, because of a strong similarity between marmoset and human genes and their functions. Based on these published reports on orthologous relationships with human P450 isoforms, inducibility, and enzymatic properties of marmoset P450 isoforms, extrapolation of results of preclinical pharmacokinetic studies to humans can be performed. Simply stated, cjESC-HLCs are ideal candidates for drug design, screening, safety and efficacy studies.

### 6.2. Phase II Enzymes

#### 6.2.1. Arylamine N-Acetyltransferases

The human genome encodes two polymorphic arylamine N-acetyltransferases (NAT1 and NAT2) and a pseudogene NATP [202]. NAT1 and NAT2 are cytosolic enzymes that catalyse the N-acetylation of arylamines, arylhydroxylamines and arylhydrazines. Even though substrate specificities of both these enzymes overlap, they are also distinct [202]. NAT1 is expressed in many tissues but NAT2 is expressed predominantly in the liver and the gut. Homologs of human NATs have been isolated from rodents [203,204], hamsters [204] and New World monkeys [205,206]. Thus far, these enzymes have not been isolated from the common marmoset. However, from our analysis of the published *C. jacchus* genome sequence [207], it appears that the marmoset genome also codes for NAT1 and NAT2 enzymes. Based on the results obtained with CYP enzymes from *C. jacchus*, one can expect a similarity between the common marmoset and human NATs for both enzymatic function and substrate specificity.

#### 6.2.2. Uridine Diphosphate Glucuronosyltransferases

Uridine diphosphate glucuronosyltransferases (UGT) are a large family of endoplasmic reticulum membrane-bound enzymes responsible for the detoxification of a wide range of xenobiotics and endogenous compounds in the Phase II drug metabolism [208]. At present, the mammalian UGT super family has 117 members, and enzymes of each family share ~40% homology, and those of sub-families share ~60% homology in their DNA sequences [209]. Among these, UGT1A proteins carry out glucuronidation of non-steroidal anti-inflammatory drugs, anticonvulsants, chemotherapeutics, steroid hormones, bile acids, and bilirubin [210]. Reduced activity of UGT1A1 caused by mutations in the *UGT1A1* gene results in progressive unconjugated hyperbilirubinemia, a rare autosomal recessive disease CN1, and patients with CN1 do eventually require a liver transplant for survival [211].

Typically, rodents and dogs are used in experimental studies as primary and secondary animal models for pharmacokinetic studies of UGT enzymes [33]. In comparison, however, human liver microsomes glucuronidate a wider range of products than the dog hepatic microsomes [212]. In contrast, human and marmoset glucuronidation in vitro is remarkably similar both quantitatively and qualitatively [212]. More recently, 11 UGTs of *UGT1A* and *UGT2B* gene families were identified and characterized in *C. jacchus* [213]. Sequence identities between human and marmosets were between 89–93% for *UGT1A* gene cluster whereas it was between 82–86% for the *UGT2B*. Among these enzymes, UGT1A4, 1A6 and 1A9 were abundantly expressed in the liver and recombinant UGT1A proteins catalyzed the glucuronidation of a variety of endobiotic and xenobiotic substrates, suggesting that they have similar molecular characteristics to that of human UGTs [213]. Therefore, the common marmoset is a useful model for studies on phase II drug metabolism.

#### 6.2.3. Other Phase II Enzymes

Other common enzymes of phase II detoxifications include sulfotransferases (SULTs), glutathione S-transferases (GSTs) and methyltransferases, such as thiopurine S-methyl transferase (TPMT) and catechol O-methyltransferase (COMT) [214]. These enzymes perform various enzymatic reactions, including glucuronidation, sulfation, methylation, acetylation, glutathione and amino acid conjugation. To date, 20 GSTs have been identified in the common marmoset [215]. Among these, a theta-class enzyme GSTT1 expressed in the liver cytosol metabolizes dichloromethane, 1-chloro-2,4-dinitrobenzene and methyl chloride at higher levels than human GSTT1 [216], while the others enzymes were able to conjugate typical GST substrates [215]. COMT catalyzes the *O*-methylation of catecholamines, estrogens and catechol-type of drugs [217]. The *C. jacchus* COMT protein is 90% identical to human COMT and is highly expressed in the liver tissues [218]. However, its substrate specificity and catalytic functions have not yet been determined. Based on our analysis of *C. jacchus*, the common marmoset genome encodes for SULTs and TPMT enzymes, and their ability to perform phase II biotransformations remains to be tested.

## 7. Future Studies and Conclusions

Over the years, the common marmoset has become an ideal large animal and NHP model to study a plethora of human diseases and pathology. By its genetic closeness to humans, it offers distinct advantages over any current rodent models, and even the Old World Monkeys, that are currently used in scientific studies. For example, since the common marmoset holds a small blood volume, immunoassays for metabolic biomarkers, such as adiponectin, leptin, ghrelin and insulin, and test compounds can be carried out in smaller volumes and relatively inexpensively [219]. High tractability, less frequent need to sedate for handling, and the use of bone marrow chimeric twins in therapy trials [20,22] are other advantages. Therefore, it has been used successfully by a number of pharmaceutical and biotechnology companies for clinical trials and toxicological testing [23,32]. However, a major drawback for *C. jacchus* is the lack of assays, antibodies and other experimental resources that are available to rodent and other NHP models.

cjESCs have a distinct advantage over their human counterparts as they are not restricted for scientific studies. cjESCs can be cultured for more than 50 population doublings, and cjESC-HLCs can be maintained in culture for a prolonged period of time without losing their phenotypic characteristics, including the expression of the asialoglycoprotein receptor, albumin, tyrosine aminotransferase, cytokeratins, CYP enzymes, and coagulation factors VII and IX [48]. High-quality cjESC-HLCs are also good substitutes for in vitro studies when compared to PHHs because they have low variability, if any, and can be easily generated in large numbers using the unlimited supply of cjESCs. Moreover, preclinical studies can be carried out with cjESCs to evaluate the safety and efficacy of hESC therapies [220], and the availability of the transgenic SCID marmoset [221] is perfect for human disease modeling, and cell transplantation due to its lack of immune responses to rejection. Furthermore, the information from the common marmoset whole genome sequence [207] can be harnessed to develop therapies that will improve human health, in part because *C. jacchus* and human genomes have >75% similarities as opposed to ~50% with rodent species.

Several groups have generated *C. jacchus* iPSCs by reprogramming skin fibroblasts [222,223], mesenchymal stem cells [224], and fetal liver cells [225]. However, they formed embryoid bodies and teratomas in immunodeficient mice, which precludes their use in cell replacement applications. Recently, HPCs from *C. jacchus* were immortalized by lentivirus-mediated expression of SV40 large T antigen [226]. Even though the hepatic differentiation potential of cj-iPSCs is unknown, cjHPCs were shown to differentiate into HLCs and cholangiocytes, both in vitro and in vivo. Isolating sufficient amounts of HPCs is a major problem because they are present in low number within the liver parenchyma; and it is only after extensive or chronic injury that overwhelms the regenerative capacity, that they are differentiated into hepatocytes [227]. Moreover, they do not survive in cell culture beyond a few population doublings. SV40-immortalized cjHPCs [226] are capable of long-term self-renewal and, therefore, can be used for in vitro studies on drug metabolism, but their use in cell replacement and tissue generation studies could be complicated by the potential risk of tumor formation in vivo. cjESCs and cjESC-HLCs are the solution for these bottlenecks as they do not suffer from these shortcomings. Moreover, hepatic differentiation of cjESCs and their propagation in culture can be carried out in perfused 3D bioreactors, and their use as 3D bioprinted spheroids has been shown for PHHs [228,229], and hiPSCs [230,231], respectively.

## Abbreviations

The following abbreviations are used in this manuscript:

11β-HSD1	11β-hydroxysteroid dehydrogenase type 1
ALD	Alcoholic liver disease
ALT	Alanine aminotransferase
AST	Aspartate transaminase
cjESC	*C. jacchus* embryonic stem cell
CYP	Cytochrome P450
CN1	Crigler-Najjar syndrome Type 1
ECM	Extracellular matrix
ESC	Embryonic stem cell
FFA	Free fatty acid
GGT	γ-glutamyl transpeptidase
HCC	Hepatocellular carcinoma
HAV	Hepatitis A virus
HBV	Hepatitis B virus
HCV	Hepatitis C virus
HDV	Hepatitis D virus
HEV	Hepatitis E virus
HF	Hepatic fibrosis
HLC	Hepatocyte-like cell
IDH	Isocitrate dehydrogenase
iPSC	Induced pluripotent stem cell
MSC	Mesenchymal stem cell
NAFLD	Non-alcoholic fatty liver disease
NASH	Non-alcoholic steatohepatitis
NHP	Non-human primate
OLT	Orthotopic liver transplantation
PH	Partial hepatectomy
PHH	Primary human hepatocyte
TAA	Thioacetamide
